# Quality of assistance provided by members of the Australian public to a person at risk of suicide: associations with training experiences and sociodemographic factors in a national survey

**DOI:** 10.1186/s12888-019-2050-6

**Published:** 2019-02-11

**Authors:** Anthony F. Jorm, Angela Nicholas, Jane Pirkis, Alyssia Rossetto, Julie-Anne Fischer, Nicola J. Reavley

**Affiliations:** Centre for Mental Health, Melbourne School of Population and Global Health, The University of Melbourne, 207 Bouverie St, Carlton, Victoria 3010 Australia

**Keywords:** Suicide, Mental health first aid, Gatekeepers

## Abstract

**Background:**

Members of the public can potentially take action to assist someone in their social network who is distressed and at risk of suicide. The present study used data from a community survey to examine training experiences and sociodemographic factors associated with the quality of assistance provided in such situations.

**Methods:**

A national telephone survey using random digit dialing was carried out with Australian adults on attitudes and intentions toward helping someone in severe distress or at risk of suicide, as well as actions taken. Participants were asked open-ended questions about their intentions to assist a hypothetical person in a vignette and about any actions they took to assist a family member or friend in distress over the previous 12 months. Each participant randomly received 1 of 6 vignettes which varied by gender and degree of suicidality portrayed. 3002 participants provided data on intentions and 932 on actions taken. Quality of Intentions and Quality of Actions were scored on 12-point scales.

**Results:**

Quality of Intentions and Quality of Actions correlated 0.28. Quality of Intentions was associated with more overt suicidality in the vignette, age 31–59 years, female gender, university education, speaking English at home, being non-Indigenous and all forms of suicide training (professional, Mental Health First Aid and other). Quality of Actions was associated with female gender, university education and other suicide training.

**Conclusions:**

Training on suicide prevention is associated with better quality of intentions and actions to help a person at risk of suicide. There are sub-groups in the population who are in greater need of such training because they have poorer quality of intentions to help and are less likely to have received training. These include males, less educated people and people from non-English speaking backgrounds.

**Electronic supplementary material:**

The online version of this article (10.1186/s12888-019-2050-6) contains supplementary material, which is available to authorized users.

## Background

People who develop a mental health problem often turn to those in their social network for support [1, 2]. The reactions they receive from others may vary from positive support to avoidance or even discrimination [3]. It is known that good social support can aid recovery [4], while negative interactions can impede it [5]. Supporters can also act to reduce suicide risk. In the case of people who die by suicide, about half communicate their intentions prior to death [6]. However, people in the social network may not feel comfortable raising the issue of suicide with the person and alerting others to the risk [7]. The ‘bystander effect’ may also inhibit action. People in the social network may also sometimes respond in a disapproving or dismissive way to expression of suicidal feelings [8], shutting down communication. However, the consensus of experts, including both professionals and people with lived experience, is that helpers should ask explicitly about suicide, assess the degree of risk and listen to the person’s suicidal feelings without judgement [9, 10].

In order to improve responses to a suicidal person, a number of gatekeeper training programs have been developed. We have previously reported data from a national survey of Australian adults on associations of training to assist a suicidal person with subsequent quality of support provided [11]. This survey involved telephone interviews with 3002 adults. Participants were presented with a vignette depicting a hypothetical person in distress and at risk of suicide. They were asked questions about their intentions to provide assistance to the person in the vignette and also about any actions they took to assist a family member or friend in distress over the previous 12 months. The questions covered 10 specific intentions and actions that were recommended in expert consensus guidelines for the public on how to assist a suicidal person, and 5 that were not recommended [9, 10]. Participants were also asked about any training they had received in how to assist a person at risk of suicide and were classified into three groups: professional training, Mental Health First Aid (MHFA) training and other training. In the previously published analyses [11], all types of training were associated with greater positive intentions and actions, and with lesser negative intentions. In particular, training was associated with a greater willingness to talk openly about suicide with a person in distress.

The previous paper analyzed responses to specific questions about intentions or actions to help a person at risk of suicide, but it did not cover other possible intentions or actions to help a person in distress. The purpose of the current article is to present further data from this survey concerning open-ended responses to questions about intentions to provide help and actual help provided over the previous 12 months. This scoring scheme is based on the action plan taught in Mental Health First Aid (MHFA) courses, which is in turn based on a series of expert consensus guidelines on how to provide mental health first aid [12]. Better quality intentions and actions are defined as those that more closely implement the action plan. The method has been used in a number of previous surveys of the quality of mental health first aid intentions and actions, including studies with Australian adults [13–15], Australian youth [16, 17], British university students [18], Sri Lankan university students [19], and Japanese high school students [20]. These studies have found that quality of intentions and actions tends to be low [13, 14, 17–19]. Better quality intentions have been found to be associated with vignettes depicting a person with depression (versus other mental disorders) [13], correct labelling of the disorder in a vignette [17, 19], female gender [13, 18], having had personal contact with a person with a mental health problem [18], lower stigma [13, 18, 19] and higher mental health literacy [13]. Better quality actions have been found to be associated with female gender [13], speaking only English at home (in an Australian sample) [15] and lower stigma [13]. Studies have also found that quality of intentions has small-to-medium correlations with quality of actions, both cross-sectionally [15] and longitudinally [15, 16].

The aims of the present study are to: (1) examine the association of training on suicide prevention with quality of intentions and actions expressed in response to open-ended questions, (2) investigate sociodemographic factors that are associated with poorer quality of intentions and actions, and with not having received training, which may be used to find sub-groups in particular need of training.

## Methods

### Participants

The survey was commissioned by *beyondblue*, which is an Australian, non-government, non-profit organization working to address issues associated with depression and anxiety disorders. The survey was conducted by Roy Morgan Research Ltd. in March 2017. The sample was drawn by a process of random digit dialing of both landlines and mobile telephones covering the whole of Australia. Up to six calls per number were made to establish contact. Interviewers ascertained whether there were residents in the household aged 18 or over and, if there were multiple, selected one for interview using the last-birthday method. Verbal consent was obtained from all respondents before commencing the interviews. Computer-assisted telephone interviews were carried out with 3002 people. There are a number of ways to calculate survey response rates. For this survey, the American Association for Public Opinion Research (AAPOR) response rate [21] was 3.1% and the simple response rate was 12.2%. AAPOR response rates in other national surveys recently run by the survey company were in the range 3.5–9.0%. Characteristics of the sample have been previously reported [11].

### Measures

The survey interview covered sociodemographic characteristics, intentions and confidence to help a person in distress, barriers and enablers to helping, actual helping behaviour, the participant’s own suicidal thoughts, help received, attitudes to suicide, exposure to suicide, training in suicide prevention and exposure to suicide prevention messages in the media. The full interview has been previously published [11]. Only the measures of specific relevance to the aims of the present paper are described in detail below.

#### Sociodemographics

Participants were asked questions about sociodemographic characteristics: gender, age group, language spoken at home, education, Indigenous status and location of residence.

#### Helping intentions

Helping intentions were assessed in relation to one of six vignettes of distressed persons that were randomly assigned to participants. The vignettes were assigned according to a 2 × 3 factorial design, with male or female versions of three scenarios: distressing life events only (“When you ask him about what is going on, John/Jenny tells you that he/she and his/her partner have separated, and he/she is having financial problems”), indirect verbal suicide communication (“John/Jenny says he/she feels he/she will never be happy again and believes his/her family would be better off without him/her”) and direct verbal suicide communication (“John/Jenny says he/she feels s/he will never be happy again and believes his/her family would be better off without him/her. You run into a friend of John’s/Jenny’s. S/he tells you that John/Jenny told him/her he/she feels desperate and has been thinking of ways to end his/her life”). The full versions of the six scenarios have been previously published [11].

Participants were then asked: “Remembering John/Jenny is someone you know well, what, if anything, would you do? Anything else?” The responses were recorded verbatim by the interviewer. The open-ended responses were scored for quality by one of the authors (JF), who was blind to other data from the survey, using the coding scheme given in Additional file 1. To ascertain inter-rater reliability, a subset of the responses was scored by another author (AFJ). The responses were scored with either 0, 1 or 2 points for quality of response to each of 6 components: approach the person, assess and assist with any crisis, listen and communicate non-judgmentally, give support and information, encourage appropriate professional help, and encourage other supports. The total score could range from 0 to 12. This scoring scheme was the same as used in previous studies [13–20], but had some additional clarifying examples added for the current study (e.g. giving specific examples of types of health professionals or sources of information the person being helped could be referred to).

Subsequently, the participants were asked to rate how likely they were to take a series of 15 specific actions, 10 of which are recommended by expert-consensus guidelines on suicide first aid, and 5 of which are not recommended [10]). These items were used to construct a 10-item Positive Intentions scale and a 5-item Negative Intentions scale, which has previously been reported on for the current sample [11]. The data on these scales are reported here as validation of the open-ended responses and to allow comparison with the data previously reported.

#### Helping behaviour

Participants were asked “In the last 12 months, has anyone in your family or close circle of friends experienced a similar level of distress to John/Jenny?” and “Did just one of your family or close friends experience this level of distress in the last 12 months, or more than one?”. If the participant knew more than one person, they were told: “Because you know more than one family member or close friend experiencing a similar level of distress, for the next few questions, I want you to think about the one you know BEST”. Participants were asked an open-ended question about what they did to help the person and the responses were recorded verbatim by the interviewer. These responses were scored for quality using the same method as for intentions to yield a score from 0 to 12. The scoring of the Quality of Actions was carried out independently of the scoring of Quality of Intentions.

Participants were then asked a series of 15 questions about specific actions taken that paralleled the questions on intentions. As for the intentions items above, the 10 recommended items were transformed into a Positive Actions scale and the 5 items recommended against were transformed into a 5-item Negative Actions scale, which has previously been reported on for the current sample [11].

#### Exposure to suicide

Participants were asked “Do you know anyone who has died by suicide?”, with responses recorded as yes or no.

#### Training received

Participants were asked “Have you ever completed any training or course in how to help someone who is suicidal?” The interviewer coded responses as professional training, MHFA, ASIST (Applied Suicide Intervention Skills Training), QPR (Question, Persuade, Refer) or other. Because of low frequencies of some types of training, these were categorized as professional, MHFA or other [11]. The commissioning organization *beyondblue* is not associated with any of the training programs evaluated in the present study.

### Statistical analysis

To assess the reliability of quality scoring of the open-ended Quality of Intentions and Quality of Actions, another author (AFJ) independently scored 50 intention responses and 50 action responses. Reliability was assessed using Krippendorff’s alpha (interval) calculated using ReCal (http://dfreelon.org/utils/recalfront/recal-oir/).

Associations among the Quality of Intentions and Quality of Actions scores and the Positive and Negative Intentions and Actions scales were assessed using Pearson correlations.

Mean scores on Quality of Intentions and Quality of Actions were compared across vignettes using 2 X 3 analyses of variance with factors of vignette gender and level of suicidality.

The factors associated with quality of intentions and actions were examined using simultaneous linear regression. The predictors were type of vignette (dummy coded) sociodemographic variables, exposure to suicide and type of training received (dummy coded). Unstandardized regressions coefficients and their 95% CIs are reported, with *P* < 0.05 used for statistical significance.

To investigate the sociodemographic factors associated with having received suicide training of any sort, a logistic regression analysis was carried out with having received training as the binary outcome and the sociodemographic factors as predictors. All analyses were carried out with IBM SPSS Statistics 22.

## Results

Sample characteristics are shown in Table 1. 3002 participants provided data that was coded for Quality of Intentions and 932 for Quality of Actions. The inter-rater reliability (Krippendorff’s alpha) for the Quality of Intentions score was 0.86, and for the Quality of Actions score it was 0.75, which was considered to be acceptable.

**Table 1:** Characteristics of the sample and allocation to vignettes (*N* = 3002)

Characteristic	*N* (%)
Female gender	1785 (59.5%)
Aged 18–30	356 (11.9%)
Aged 31–59	1408 (46.9%)
Aged 60+	1238 (41.2%)
Bachelor’s degree or above	1215 (40.5%)
Non-urban location	1254 (41.8%)
Exposed to suicide	1839 (61.3%)
Professional suicide training	256 (8.5%)
Mental Health First Aid training	141 (4.7%)
Other suicide training	221 (7.4%)
Distressing life events only vignette: Male	511 (17.0%)
Distressing life events only vignette: Female	529 (17.6%)
Indirect verbal suicide communication vignette: Male	492 (16.4%)
Indirect verbal suicide communication vignette: Female	455 (15.2%)
Direct verbal suicide communication vignette: Male	497 (16.6%)
Direct verbal suicide communication vignette: Female	518 (17.3%)

Table 2 shows the mean scores on Quality of Intentions and Quality of Actions, which were low for all of the six vignettes. Quality of Intentions differed by level of suicidality in the vignettes (*P* < 0.001), with scores increasing as suicidality was more overt, but there was no effect of gender of the person in the vignette. For Quality of Actions there were no differences between vignettes.

**Table 2:** Quality of intentions and actions by type of vignette: mean (SD), 95% CI and N

Type of Vignette	Quality of Intentions			Quality of Actions		
	Mean (SD)	95% CI	*N*	Mean (SD)	95% CI	*N*
Distressing life events only: Male	2.60 (1.33)	2.49–2.72	511	2.77 (1.38)	2.56–2.97	175
Distressing life events only: Female	2.57 (1.29)	2.46–2.68	529	2.80 (1.50)	2.57–3.02	176
Indirect verbal suicide communication: Male	3.00 (1.49)	2.87–3.13	492	2.49 (1.56)	2.23–2.75	145
Indirect verbal suicide communication: Female	3.18 (1.42)	3.05–3.32	455	2.72 (1.36)	2.51–2.92	165
Direct verbal suicide communication: Male	3.22 (1.46)	3.10–3.35	497	2.66 (1.30)	2.43–2.89	125
Direct verbal suicide communication: Female	3.23 (1.51)	3.10–3.36	518	2.55 (1.33)	2.33–2.77	146

Table 3 shows the correlations between the Quality of Intentions and Quality of Actions scores, and correlations of these scores with the Positive and Negative Intentions and Actions scales. There was a medium association between Quality of Intentions and Quality of Actions. There were also medium correlations between the Quality of Intentions and the Positive Intentions scale, and between the Quality of Actions and the Positive Actions scale. Other correlations were small.

**Table 3:** Correlations of Quality of Intentions and Quality of Actions with each other and with Positive and Negative Intentions and Actions scales (data pooled across vignettes)

	Quality of Intentions	Quality of Actions
Quality of Actions	0.28*	1.00
Positive Intentions Sale	0.24*	0.16*
Negative Intentions Scale	−0.08*	−0.10*
Positive Actions Scale	0.06*	0.28*
Negative Actions Scale	−0.08*	0.06

*P < 0.05

The binary logistic regression analysis on sociodemographic factors associated with training of any type showed that training was more likely to have been received by females (OR = 1.22, 95% CI = 1.00–1.49, *P* = 0.046), those with a bachelor degree or above (OR = 2.05, 95% CI = 1.68–2.50, *P* < 0.001) and those exposed to suicide (OR = 1.88, 95% CI = 1.52–2.33, P < 0.001), while it was less likely to have been received by people who use a language other than English (OR = 0.58, 95% CI = 0.37–0.91, *P* = 0.02).

Multiple linear regression analyses were carried out to assess whether type of training predicted intention and action scale scores. The unstandardized coefficients after adjustment for type of vignette presented are shown in Table 4. Better Quality of Intentions was significantly associated with age 31–59 years, female gender, higher education and all forms of training. Quality of Actions was significantly associated with a smaller set of predictors: female gender, higher education and other suicide training. Consistent with the data shown in Table 2, the quality of intentions was greater when there was more overt suicidality in the vignette, whereas quality of actions taken was not associated with vignette characteristics.

**Table 4:** Predictors of quality of intentions and actions: Unstandardized regression coefficients (and 95% CIs)^1^

Predictors	Quality of Intentions	Quality of Actions
Age		
18–30	Reference	Reference
31–59	0.20 (0.04, 0.36)*	0.20 (−0.07, 0.46)
60+	−0.01 (− 0.17, 0.16)	−0.08 (− 0.37, 0.21)
Female gender	0.52 (0.42, 0.62)*	0.36 (0.16, 0.54)*
Language other than English	−0.29 (− 0.48, − 0.10)*	−0.24 (−64, 0.16)
Bachelor’s degree or higher	0.30 (0.19, 0.40)*	0.47 (0.28, 0.66)*
Non-urban location	−0.06 (− 0.16, 0.04)	−0.05 (− 0.24, 0.13)
Indigenous	− 0.52 (− 0.88, − 0.15)*	−0.18 (− 0.67, 0.30)
Exposed to suicide	0.04 (− 0.06, 0.14)	0.04 (− 0.16, 0.23)
Professional training in suicide	0.28 (0.10, 0.46)*	0.01 (−0.30, 0.32)
Mental Health First Aid training	0.47 (0.23, 0.69)*	0.30 (−0.07, 0.67)
Other suicide training	0.51 (0.32, 0.70)*	0.48 (0.17, 0.79)*
Vignette characteristics^a^		
Distressing life events: Female	−0.02 (− 0.18, 0.15)	0.05 (− 0.23, 0.34)
Indirect suicide communication: Male	0.38 (0.21, 0.54)*	− 0.24 (− 0.54, 0.07)
Indirect suicide communication: Female	0.53 (0.36, 0.70)*	− 0.10 (− 0.39, 0.20)
Direct suicide communication: Male	0.64 (0.47, 0.81)*	− 0.12 (− 0.43, 0.20)
Direct suicide communication: Female	0.62 (0.45, 0.78)*	− 0.21 (− 0.51, 0.09)
Constant	2.32 (1.82–2.82)	2.30 (1.45, 3.12)
Adjusted R^2^	0.12	0.08

^a^Reference group is ‘Distressing life events: Male’; *P < 0.05

## Discussion

This study examined whether training on suicide and sociodemographic factors were associated with better quality intentions and actions in open-ended responses to a vignette. After adjusting for type of vignette and sociodemographic factors, all types of training (professional, MHFA and other) were associated with Quality of Intentions, while only ‘other training’ was significantly associated with Quality of Actions. The difference in findings may relate to statistical power, as only a third of the sample reported on actions taken to assist someone in the previous 12 months.

These findings support the previous analyses from the current sample showing that training was associated with greater positive intentions and actions in relation to suicide risk, and with lesser negative intentions [11]. However, the current findings show a broader association with helping intentions, as the responses were scored for quality of responses to mental health problems in general, rather than specifically to suicide risk. The findings on mental health first aid intentions are consistent with the results from randomized controlled trials of MHFA training, which have found improvements in intentions to assist a person with a mental health problem at 6-month and longer follow-up [22], but extend these to everyday conditions outside of a trial context.

The findings on sociodemographic associations point to sub-groups in the population that may be in particular need of intervention. Quality of Intentions was poorer in males, young people, older people, those without a university degree, non-English speakers and Indigenous people. Males and less educated people also had poorer Quality of Actions. While it is possible that these sub-groups were less able to express their intentions and actions in response to open-ended questions, this seems less likely when the responses were given orally and recorded by the interviewer. Furthermore, the data on predictors of having received training also showed that males, less educated people and non-English speakers were less likely to have had any type of suicide training, which also indicates a need for greater attention to these sub-groups.

There was a correlation of 0.28 between Quality of Intentions and Quality of Actions. This association is comparable to what has been found in previous studies with these measures. In studies of adults, Rossetto et al. [15], found correlations of 0.31 cross-sectionally and 0.27 longitudinally, while Rossetto et al. [14] found a correlation of 0.20 cross-sectionally. In a study of youth, Yap et al. [16] reported a standardized beta of 0.13 longitudinally. There are a number of factors that may reduce the correlation between intentions and actions. The first is error of measurement, which is reflected in the imperfect inter-rater reliabilities of the quality ratings. A second factor is that the intentions were rated in relation to a vignette which varied in expression of suicidality, whereas the actions were rated in relation to a real situation where the person involved may have had a different degree of suicidality to the vignette. A third factor is that the provision of mental health first aid in real life involves a number of factors that are not present in a hypothetical vignette. Rossetto et al. [23] have presented a model of help giving towards people with mental health problems, which describes the complex factors that may determine whether any action is taken and what is done. These include the triggers of concern that are present (e.g. whether the person approaches the helper, knowledge of the person’s history), the considerations that inform decisions to help (e.g. relationship to the person, whether others are available to help, perceived danger) and the recipient’s reaction to any help provided (e.g. acceptance or resistance to help, improvement or worsening of symptoms). Such factors indicate the need for training programs to consider how to overcome potential barriers to action in addition to training people how best to respond to a suicidal person.

The limitations of the survey have been previously discussed [11]. The data are cross-sectional, which limits causal inference. There is limited information on the type of training received and no data on how long ago it occurred. The low response rate means that the sample may not be representative of the population. There may also be limitations in recall of helping actions, which would affect the validity of the Quality of Action responses. The open-ended nature of the responses may have affected associations with sociodemographic characteristics, in particular with non-English speaking background.

## Conclusions

The findings show that training on suicide prevention is associated with better quality of intentions and actions to help a person at risk of suicide, with non-professional training being associated with similar effects to professional training. Such non-professional training has had substantial uptake in Australia, but could feasibly be extended considerably given that the training infrastructure is available, thereby increasing national capacity to support suicidal persons who may not be in contact with professional services. However, there are sub-groups in the population who are in greater need of such training because they have poorer quality of intentions to help and are less likely to have received training. These include males, less educated people and people from a non-English speaking background.

## Additional file

Additional file 1: Scoring criteria for quality of intentions and quality of actions for open-ended responses to a distressed person at risk of suicide. (DOCX 36 kb)

## Abbreviations

ASIST: Applied Suicide Intervention Skills Training
MHFA: Mental Health First Aid
QPR: Question, Persuade, Refer
SPSS: Statistical Package for the Social Sciences

## References

[1] Jorm AF, Christensen H, Medway J, Korten AE, Jacomb PA, Rodgers B (2000). Public belief systems about the helpfulness of interventions for depression: associations with history of depression and professional help- seeking. Soc Psychiatry Psychiatr Epidemiol.

[2] Jorm AF, Griffiths KM, Christensen H, Parslow RA, Rogers B (2004). Actions taken to cope with depression at different levels of severity: a community survey. Psychol Med.

[3] Reavley NJ, Jorm AF (2015). Experiences of discrimination and positive treatment in people with mental health problems: findings from an Australian national survey. Aust N Z J Psychiatry.

[4] Keitner GI, Ryan CE, Miller IW, Kohn R, Bishop DS, Epstein NB (1995). Role of the family in recovery and major depression. Am J Psychiatr.

[5] Hooley JM (2007). Expressed emotion and relapse of psychopathology. Annu Rev Clin Psychol.

[6] Pompili M, Belvederi Murri M, Patti S, Innamorati M, Lester D, Giradi P, Amore M (2016). The communication of suicidal intentions: a meta-analysis. Psychol Med.

[7] Owens C, Owen G, Belam J, Lloyd K, Rapport F, Donovan J, Lambert H (2011). Recognising and responding to suicidal crisis within family and social networks: qualitative study. BMJ.

[8] Sweeney L, Owens C, Malone K (2015). Communication and interpretation of emotional distress within the friendships of young Irish men prior to suicide: a qualitative study. Health Soc Care Community.

[9] Ross AM, Kelly CM, Jorm AF. Re-development of mental health first aid guidelines for suicidal ideation and behaviour: a delphi study. BMC Psychiatry. 2014;14(1). 10.1186/s12888-014-0241-8 PMC4199061 25213799

[10] Nicholas A, Rossetto A, Jorm A, Pirkis J, Reavley N. Importance of messages for a suicide prevention media campaign. Crisis. 2018:1–13. 10.1027/0227-5910/a000517 29618269

[11] Jorm AF, Nicholas A, Pirkis J, Rossetto A, Reavley NJ (2018). Associations of training to assist a suicidal person with subsequent quality of support: results from a national survey of the Australian public. BMC Psychiatry.

[12] Jorm AF, Ross AM (2018). Guidelines for the public on how to provide mental health first aid: a narrative review. BJPsych Open.

[13] Rossetto A, Jorm AF, Reavley NJ. Examining predictors of help giving toward people with a mental illness: results from a national survey of Australian adults. SAGE Open. 2014;4. 10.1177/2158244014537502.

[14] Rossetto A, Jorm AF, Reavley NJ. Quality of helping behaviours of members of the public towards a person with a mental illness: a descriptive analysis of data from an Australian national survey. Ann General Psychiatry. 2014;13:2. 10.1186/1744-859X-13-2 PMC3898824 24438434

[15] Rossetto A, Jorm AF, Reavley NJ (2016). Predictors of adults' helping intentions and behaviours towards a person with a mental illness: a six-month follow-up study. Psychiatry Res.

[16] Yap MBH, Jorm AF (2012). Young people's mental health first aid intentions and beliefs prospectively predict their actions: findings from an Australian National Survey of youth. Psychiatry Res.

[17] Yap MBH, Reavley NJ, Jorm AF (2015). Is the use of accurate psychiatric labels associated with intentions and beliefs about responses to mental illness in a friend? Findings from two national surveys of Australian youth. Epidemiol Psychiatr Sci.

[18] Davies EB, Wardlaw J, Morriss R, Glazebrook C (2016). An experimental study exploring the impact of vignette gender on the quality of university students' mental health first aid for peers with symptoms of depression. BMC Public Health.

[19] Amarasuriya SD, Reavley NJ, Rossetto A, Jorm AF (2017). Helping intentions of undergraduates towards their depressed peers: a cross-sectional study in Sri Lanka. BMC Psychiatry.

[20] Yoshioka K, Reavley NJ, Rossetto A, Jorm AF (2015). Beliefs about first aid for mental disorders: results from a mental health literacy survey of Japanese high school students. Int J Cult Ment Health.

[21] Research AAfPO (2016). Standard definitions: final dispositions of case codes and outcome rates for surveys.

[22] Morgan AJ, Ross A, Reavley NJ (2018). Systematic review and meta-analysis of mental health first aid training: effects on knowledge, stigma, and helping behaviour. PLoS One.

[23] Rossetto A, Jorm AF, Reavley NJ (2018). Developing a model of help giving towards people with a mental health problem: a qualitative study of mental health first aid participants. Int J Ment Heal Syst.

