# Supplementary material for: Quality of assistance provided by members of the Australian public to a person at risk of suicide: associations with training experiences and sociodemographic factors in a national survey
Source: BMC Psychiatry. 2019 Feb 11;19:68. doi: 10.1186/s12888-019-2050-6 (PMC6371420; doi:10.1186/s12888-019-2050-6)
Supplement: Supplementary file 1 — Scoring criteria for quality of intentions and quality of actions for open-ended responses to a distressed person at risk of suicide. (DOCX 36 kb) [file 12888_2019_2050_MOESM1_ESM.docx]

Additional file 1: Scoring criteria for quality of intentions and quality of actions for open-ended responses to a distressed person at risk of suicide.

General scoring criterion: If response is to give the acronym “ALGEE”, but nothing else, give 1 point per action, i.e. total of 6. Only give extra points for an action where specific detail is given.

| **Component of mental health first aid response** | **0 points**  **(no mention or inadequate response)** | **1 point**  **(superficial)** | **2 points**  **(specific detail)** |
| --- | --- | --- | --- |
| **A** *(approach the person)* | No mention of how to approach the person | Mentions any one of the following:   - Before making an approach, sought information for myself on what to do to help the person (e.g. went to a GP/health professional; mental health website or Helpline). - Considering or making an approach or engaging the person/or if they don’t wish to talk to me, finding someone more suitable. - The setting (e.g. choose a suitable time or place/took them for a coffee/meal/quiet place to talk/outside of work/in private). - The content of the approach (e.g. let the person know that you are available to talk/ don’t put pressure on the person to talk right away/if they don’t want to talk now, they can at another time when they are ready; let the person know your concerns (e.g. you seem depressed/you are not your usual self/I’ve noticed some changes in you); state the specific behaviours you are concerned about). | Mentions **more than one** of the following:   - Before making an approach, sought information for myself on what to do to help the person (e.g. went to a GP/health professional; mental health website or Helpline). - Considering or making an approach or engaging the person/or if they don’t wish to talk to me, finding someone more suitable. - The setting (e.g. choose a suitable time or place/took them for a coffee/meal/quiet place to talk/outside of work/in private). - The content of the approach (e.g. let the person know that you are available to talk/ don’t put pressure on the person to talk right away/ if they don’t want to talk now, they can at another time when they are ready; let the person know your concerns (e.g. you seem depressed/you are not your usual self/I’ve noticed some changes in you); state the specific behaviours you are concerned about). |
| **A** *(assess and assist with any crisis)* | Assessment/observation of risk or dealing with crises not mentioned | Mentions assessing/observing risk or dealing with crises, but no details are given about what risks are assessed or crises dealt with. Examples include: called ambulance/emergency services/Police or CATT team. | Mentions specific risks that are assessed/observed or crises dealt with, or actions taken to deal with the crises (e.g. remove means of harm/padlocked tablets in the house).  Or mentions any of the following:   - Suicidal thoughts and behaviours (e.g. wanted to kill themselves/asked if had plans for suicide) - Non-suicidal self-injury - Panic attacks - Traumatic events - Severe psychotic states - Severe effects of alcohol misuse - Severe effects of drug misuse - Aggressive behaviours |
| **L** *(listen and communicate non-judgementally)* | Listening or talking to person not mentioned, or negative type of interaction mentioned, e.g. “Confront them about it”  When conversation is one-way, e.g. “..*tell* them *my* concerns”, score this as zero. Needs to be two-way, e.g. “talk” | Mentions talking and/or listening to the person, but no details are given about the quality of the interaction, e.g. Talk to him/listen to problems/ discussed problems. | Mentions talking and /or listening, but also gives some specification of how this should be done or the quality of the interaction. Examples include:   - Listen empathically/listen as a friend/build rapport - Accept the person/not judging, being critical, or expressing frustration - Validate feelings/experience - Not being confrontational/use neutral non-verbal skills such as open stance or comfortable eye contact - Have a positive attitude - Keep calm |
| **G** *(give support and information)* | Support and information not mentioned | Mentions **one** type of giving support or information from one of the following categories:   - Emotional/social support “You’ll be okay/ professional help is available/ give hope for recovery/ I’m here for you.” Acknowledge as a real problem/ normalise/empathize with how they feel. Give reassurance/ spend time with them/ think of ideas/brainstorm options/help them draw on past coping mechanisms. Provide ongoing support/ follow-up/monitor/check back in to see how they are going/stayed in contact. Share own experience of mental illness and help-seeking. - Practical help with tasks: including accompanying to professional appointment; helped financially; helped them transition to new accommodation after hospital/find suitable nursing home/moved in with me; organized after care; made them meals; was their Advocate; provided them with a mentor who was culturally appropriate.   *Work related*: cover for person at work while they are unwell; talked to their employer/ guided them back to their work or helped them obtain work. (If respondent is boss or supervisor and changed tasks for distressed person, code as give support).   - Offers resources or information, such as links, guidelines or fact-sheets on mental health related topics/gambling or local community services/charities/Salvos/ Centrelink/financial support services.     N.B. While *giving information* about health professionals is scored here, don’t count *recommending or suggesting* professionals (as this is ‘E’- encourage appropriate professional help). | Mentions giving support or information from **more than one** of the following categories:   - Emotional/social support “You’ll be okay/ professional help is available/ give hope for recovery/ I’m here for you.” Acknowledge as a real problem/ normalise/empathize with how they feel. Give reassurance/spend time with them/ think of ideas/brainstorm options/help them draw on past coping mechanisms. Provide ongoing support/ follow-up/monitor/check back in to see how they are going/stayed in contact. Share own experience of mental illness and help-seeking. - Practical help with tasks: including accompanying to professional appointment; helped financially; helped them transition to new accommodation after hospital/find suitable nursing home/moved in with me; organized after care; made them meals; was their Advocate; provided them with a mentor who was culturally appropriate.   *Work related*: cover for person at work while they are unwell; talked to their employer/ guided them back to their work or helped them obtain work. (If respondent is boss or supervisor and changed tasks for distressed person, code as give support).   - Offers resources or information, such as links, guidelines or fact-sheets on mental health related topics/gambling or local community services/charities/Salvos/Centrelink/financial support services. |
| **E** *(encourage appropriate professional help)* | Professional or getting outside help not mentioned; or negative methods of accessing outside help mentioned, i.e. force | Mentions professional help/getting outside help (whether encourage/refer/engage or just ‘help/outside help’), but does not specify type of help, e.g. “I’d take her to a professional” or “I’d seek professional support”. | Mentions recommending or referring to specific type of help, or specifies ‘appropriate’ professional help. Specific types of help are:   - GP/Psychiatrist/Psychologist/ Social Worker/Counsellor/Youth Worker/ Occupational Therapist/School Counsellor/ Mental Health Nurse/Dietician or Nutritionist - Mental health professional/Health professional - Medical professional/ medical assistance/ health services, “I’d seek medical assistance.” - Antidepressants (i.e. made sure they took their meds) - ‘Specialist’ - Counselling/therapy - Clinic e.g. Headspace - Hospital (if potential crisis)/Residential facility - Rehab/detox centre - Helpline e.g. Lifeline, beyondblue, crisis line - Employee Assistance Program (EAP/support plan) |
| **E** (*encourage other supports)* | Other supports not mentioned, or potentially harmful strategies mentioned, e.g. use alcohol or other drugs, natural medicines such as homeopathy and naturopathy that are **not** evidence-based supports.    YMHFA specific: mentions consulting adolescent’s friends to obtain information about adolescent | - Mentions encouraging or asking about **one** type of support only, from the following: - A specific self-help strategy/program - Seeking support from family or friends “..talked to parents and gave them ideas on what they could do..”; “discussed with friends ways we could help him/her..” If a minor, encourage speaking to their parent or a responsible adult. - Seeking support from others who have had similar experience/mental health or other support group, e.g. AA, support group for new mums, MensLine. - Church-group/Minister/Pastor - Seeking support from the school (teachers, wellbeing coordinator, etc). - Return to work strategies: speak with HR/Manager/Supervisor about workplace adjustments (e.g. time off to sort things out/flexi-hours/altered duties). - Self-help or complementary therapies that **are** evidence based (e.g. exercise, meditation, light therapy, massage, brain foods such as omega-3). - If a victim of crime, report criminal matters to police. | Mentions encouraging or asking about a type of support from **more than one** of the following categories. These include:   - A specific self-help strategy/program - Seeking support from family or friends “..talked to parents and gave them ideas on what they could do..”; “discussed with friends ways we could help him/her..” If a minor, encourage speaking to their parent or a responsible adult. - Seeking support from others who have had similar experience/mental health or other support group, e.g. AA, support group for new mums, MensLine. - Church-group/Minister/Pastor - Seeking support from the school (teachers, wellbeing coordinator, etc). - Return to work strategies: speak with HR/Manager/Supervisor about workplace adjustments (e.g. time off to sort things out/flexi-hours/altered duties). - Self-help or complementary therapies that **are** evidence based (e.g. exercise, meditation, light therapy, massage, ‘brain foods such as omega-3). - If a victim of crime, report criminal matters to police. |
